# Marketing strategies used for alternative protein products sold in Australian supermarkets in 2014, 2017, and 2021

**DOI:** 10.3389/fnut.2022.1087194

**Published:** 2022-12-22

**Authors:** Paige G. Brooker, Gilly A. Hendrie, Kim Anastasiou, Rachel Woodhouse, Theresa Pham, Michelle L. Colgrave

**Affiliations:** ^1^Health and Biosecurity, Commonwealth Scientific and Industrial Research Organisation (CSIRO), Adelaide, SA, Australia; ^2^Department of Nutrition and Dietetics, Flinders University, Bedford Park, SA, Australia; ^3^Health Research and Innovation, The National Heart Foundation of Australia, Docklands, VIC, Australia; ^4^Grains & Legumes Nutrition Council, North Sydney, NSW, Australia; ^5^Agriculture and Food, Commonwealth Scientific and Industrial Research Organisation (CSIRO), Saint Lucia, QLD, Australia

**Keywords:** front-of-pack labeling, alternative protein, plant-based, food marketing, vegetarian, flexitarian

## Abstract

**Introduction:**

Marketing plays an important role in consumers’ perceptions and acceptance of new foods. The purpose of this study was to investigate the marketing strategies used for alternative protein products available in Australia in 2014, 2017, and 2021.

**Methods:**

Product data were extracted from FoodTrack*™*, an established database of packaged supermarket products. Marketing strategies investigated included product format descriptors, front of pack (FOP) labeling claims, price, and in-store placement (2021 only).

**Results:**

Data from 292 alternative protein products (*n* = 12 tofu-based products; *n* = 100 legume-based products; and n = 180 plant-based meats) were analyzed. Across the product range, “burgers” (*n* = 86), “strips and similar” (*n* = 51) and “sausages” (*n* = 42) were the most common product formats, accounting for ∼61% of the product range. Nutrient content claims featured on 273 (93%) products. “Positive” nutrient claims (those highlighting the presence of a nutrient) occurred on FOP labels four times more than “negative” nutrient claims (those highlighting the absence or low levels of a nutrient; 432 versus 101, respectively). Protein-related claims were the most common “positive” nutrient claim (*n* = 180, 62%). Health claims on FOP labels appeared on 10% of products. Most products (*n* = 265, 91%) mentioned a dietary pattern (such as “vegetarian” and “plant-based”), or a combination of dietary patterns on their FOP label. The price of alternative products increased over time; between 2014 and 2021, on average, the unit price increased (9% increase, *p* = 0.035) and the pack size decreased (14% decrease, *p* < 0.001). There was inconsistency in product placement across the eight stores visited. Occasionally (*n* = 3 of 13 locations), chilled alternative protein products were positioned near conventional meat products. More commonly, alternative protein products shared space with other vegetarian products (such as non-dairy cheeses and tofu blocks) or alongside convenience products, suggesting these products are promoted as convenience foods, or options for individuals with special dietary needs.

**Discussion:**

This study provides a useful evidence base to understand the marketing strategies used for alternative protein products. It appears from this analysis that considerable effort has gone into providing consumers with a level of familiarity and comfort prior to purchasing these alternative protein products.

## 1 Introduction

Consumers are shifting toward more plant-based dietary patterns, driven by environmental, health and animal welfare concerns (1–3). In, one in three Australians were consciously limiting their meat consumption to some extent (4, 5). Alternative protein sources are being explored by consumers and the food industry as possible substitutes or complementary sources of protein to conventional animal-based proteins. They are usually derived from soy or other plant-based protein-rich foods such as legumes, and sold in various formats including burger patties, sausages, stir fry cubes, and mince (6). While the role of these products in healthy and sustainable diets is contested, they are becoming more commonplace on supermarket shelves (7, 8). According to estimates from the Mintel Global New Product Database, since 2015, the number of plant-based meats has exceeded 4,400 different products globally (from Mintel Group Ltd., in Estell et al., (3)). However, consumers who regularly buy these products are still the exception (3, 9, 10).

The way products are marketed can influence consumers’ perceptions and acceptance of new foods, including alternative protein products (11), and plays an important role in their food purchase decisions. The “marketing mix” includes a range of factors that are considered when marketing a product including consideration of what consumers want, how the product is perceived, and how it stands out from other products. For food products, this includes factors such as product descriptors and images available on the package, the price, as well as in store placement and promotion.

Front of pack (FOP) labels provide a place to market products, and they have been shown to affect liking ratings of foods, as well as individual’s intention to buy foods (12). Martin et al. (13) investigated consumer purchasing preferences and willingness to pay for plant-based versus meat-based sausages in a sample of 102 “regular and occasional” consumers of pork. Based on (blind) taste alone, consumers preferred, and were more willing to pay for, meat- (pork) based sausages. However, when consumers were presented with the products’ packaging and additional information related to consequences/benefits for health and the environment (favoring plant-based products), consumer’s willingness to purchase plant-based sausages was significantly increased and was similar to their willingness to purchase meat-based sausages. Another study investigated the effect of a vegetarian label on calorie perception and food choices. Vegetarian products were perceived to be less energy-dense than their non-vegetarian equivalents, however, participants did not report an increase in their intention to eat more vegetarian products when the label was present (14). These findings suggest that providing consumers with additional product information influences their perception, preference and purchase decisions around plant-based products.

Consumers use food labels to understand how to prepare or consume products, as well as to obtain information regarding their healthfulness, food safety and origin (15). However, the recent increase in the use of some types of health-related food labeling by manufacturers to emphasize the positive nutritional attributes of their products has concerned some health professionals. The presence of a health-related claim may have a halo effect, whereby consumers perceive products carrying a claim to be healthier than those without (16). Health halos may also result in “spill-over” effects, whereby products labeled with one claim are assumed to have other (non-claimed) nutrition or health benefits (17). For example, there is evidence to suggest the “organic” label influences consumer perception of the products nutritional quality (18). Findings from a recent meta-analysis suggest that the presence of health or nutrition claims on FOP labels also influences the time consumers spend evaluating the nutrition information panel. In the presence of a claim, such as “low fat,” on the FOP label, consumers spend less time evaluating the nutritional facts, and are more likely to base their purchasing decision primarily on the label (19). Lacy-Nichols et al. (20) investigated the presence and frequency of health and nutrition claims made on FOP labels and websites of 216 alternative protein products sold across 16 manufacturers in the USA. Most nutrient claims were for nutrients usually associated with meat; 94% of products carried a protein claim, and 30% carried a cholesterol claim. No health claims were included on the FOP labels of these alternative protein products, which is interesting given the perceived healthfulness of this product range. Little is known about the prevalence and types of claims made on the FOP labels of alternative protein products in Australia.

Convenience and familiarity are also important purchasing drivers for novel foods, including alternative protein products (21–26). Subsequently, plant-based meats are often presented in more convenient pre-prepared formats, and in formats that are familiar like conventional meat products, and intended for use in customary meals and recipes, as well as social settings such as barbeques (23, 27). In their audit of plant-based meats sold in Australian supermarkets in, Curtain and Grafenauer (28) found these products were most commonly presented as burgers, followed by sausages and chicken. “Other” products (such as bacon and deli slices), mince and seafood were also available, but were fewer in number. The authors also report anecdotal evidence that plant-based meat products are often “*placed in the chilled meat section adjacent to meat, many with a clear window as part of the packaging so the appearance and similarity to meat is in view”* (28) *(p. 2611).* Product placement “cross-merchandising” strategies have been used to influence customers’ food purchasing decisions (29, 30). Piernas et al. (31) evaluated the weekly sales of meat and meat-free products from 108 stores in the United Kingdom over 123 weeks. Repositioning meat-free products into the meat aisle (intervention stores) significantly increased the sale of meat-free products, with no impact on sales of meat products when compared with matched control stores (meat-free products remained in their original location, usually in their own meat-free section outside the meat aisle).

Price is another key product-related attribute often associated with consumer acceptance of new products, including alternative protein products (32, 33). Findings from a consumer survey of 1,000 US adults suggest that the likelihood of trying an alternative protein product increased with income. Survey respondents who earned over 120,000 USD were the most likely consumers, and those making less than 40,000 USD were the least likely consumers (34). In a hypothetical choice experiment consumers were given the option of purchasing burgers made from animal-based or plant-based protein. Willingness to purchase products was tested against several factors including price, taste, health, environmental impact, and animal welfare. Individuals with a higher preference for alternative protein products were less sensitive to price (35). Reaching price parity, such that alternative protein products cost either the same or less than conventional animal equivalent products is an important component of consumer acceptance of these products (36).

Previous studies which have investigated the marketing strategies used for alternative protein products have focused exclusively on one aspect the “marketing mix” (i.e., one of: product, price, promotion or place) (20, 28, 31, 37, 38), and only one study has been conducted in Australia (28). Therefore, the purpose of this study was to explore the suite of marketing strategies used for alternative protein products, including tofu- and legume-based “convenience” products, and plant-based meats, and to determine if these marketing strategies have changed over time. Specifically, this study focused on the use of nutrition and health claims on products’ FOP labels, descriptors used to communicate the products format, and the price of alternative protein products available in Australian supermarkets in 2014, 2017, and 2021. To investigate the promotional strategies used by retailers, a cross-sectional analysis of the in-store location of products, cross-merchandising and other in-store displays was conducted for products collected in 2021.

## 2 Materials and methods

Data for alternative protein products available in Australian supermarkets were obtained from the FoodTrack™ database; an Australian database for packaged food and beverages developed by the Commonwealth Scientific and Industrial Research Organisation (CSIRO) and the National Heart Foundation of Australia in 2014. It is updated on an annual cycle using information from supermarkets in metropolitan Victoria. With permission from local or national store managers, trained data collectors visited major retailers in Australia: Woolworths (since 2014), Coles (since 2014), ALDI (since 2016) and IGA (since 2017) supermarkets, and used a customized App with barcode recognition software to collect product information and images of products for sale (39). To allow for a comprehensive assessment of products available, each supermarket was visited twice (i.e., eight stores are visited in total). To understand the changes over time, three time points were chosen (2014 the first year of data collection; 2017 a midpoint; and 2021 the most recent data collection year).

For this study, products collected under the “Vegetarian-processed” grocery category were analyzed. This category includes alternative protein products that are positioned as alternatives to processed meat products. This includes those products which are designed to be included as part of a meal as more traditional alternatives to meat (e.g., tofu blocks and textured vegetable protein requiring reconstitution) as well as products designed to be convenient, direct substitutes to conventional processed meat products (e.g., burgers, sausages, falafel, schnitzels, and deli slices). Vegetarian ready-made meals, such as vegetarian lasagna, along with canned and dried legumes, and products with pastry, such as vegetarian pies and pastries, were not collected under the Vegetarian-processed grocery category in FoodTrack™ and were excluded from this analysis.

The purpose of this study was to examine the range of alternative protein products positioned as a direct replacement for conventional meat (“convenience products”). Therefore, products were grouped into four subcategories, based on their format and primary source of protein, according to study definitions. These were: (i) Tofu; (ii) Processed legumes; (iii) Meat analogs; and (iv) Other (Table 1). Products that were considered to be ingredients and included as part of a meal (i.e., tofu – block and pieces, and textured vegetable protein) were excluded from the analysis.

**TABLE 1:** Description of the subcategories for products collected in the Vegetarian-processed grocery category used in this study.

Subcategory	Definition
**Tofu**	
Tofu blocks or pieces*	All soybean/bean curd products including both plain (silken, firm, organic) and flavored tofu/tempeh (sweet chili, teriyaki)
Tofu products	Soybean-/tofu-based products made into different formats (e.g., tofu sausages)
**Processed legumes**	
Legume products	Legume-based products (whole ingredient combinations, or kibbled/flour) including lentil burgers, falafel mixes/ready-made falafel.
**Meat analogs**	
Textured vegetable protein*	Textured vegetable protein – plain or flavored with spices, requiring reconstitution
Plant-based meats	Plant-based alternatives to processed meat products typically prepared as sausages, mince, strips, nuggets, burgers, hot dogs, slices, meatballs, fillets, bacon, pepperoni, luncheon meat, schnitzels, patties, nutmeat and other Vegetarian-processed meat and smallgoods equivalents. Products are often made from textured (or hydrolysed) vegetable protein, soy, pea protein, mycoprotein, wheat gluten.
**Other**	
Nil*	Products which do not contain tofu/soybean, legumes, or plant protein (e.g., vegetable patties, canned jackfruit)

Products were grouped into their respective subcategory based on their primary source as per their ingredient listing: tofu, tofu/soybean; traditional meat alternatives, legumes (whole, kibbled, or as flour); or meat analogs, vegetable protein/wheat gluten. Products which did not contain any of the forementioned ingredients were considered “nil”.

*Indicates products which were excluded from analysis.

### 2.1 Data extraction and synthesis

Information for all alternative protein products in the FoodTrack™ database, including manufacturer and brand details, product description, pack and serving sizes, and pricing information were exported to Microsoft Excel^®^ (version 2018). Photographs of the FOP labels for all eligible products were individually inspected, and text from the label were transcribed into Microsoft Excel^®^ (version 2018). Text presented as a logo (e.g., “Australian made,” and “Non-GMO Project Verified”) were also transcribed. Text describing the storage or preparation time (e.g., “freezer friendly,” or “ready in 10 min”) were not extracted, nor were data from nutrition information panels and ingredients listings (where they appeared on the FOP label). Where available, products’ country of origin was recorded from images of product packaging in FoodTrack™ and supplemented with online searches of brand/manufacturer/retailer’s website where necessary.

#### 2.1.1 Product format

Products were grouped into common categories based on the format they took, as described by their product name or on the FOP label. The categories were chosen for comparability to other literature (28, 38) and included burgers, sausages, meatballs, mince, smallgoods, strips, chunks, falafel and an additional “other” category with products that fell outside of these categories (see Table 2).

**TABLE 2:** Classification of categories of product format.

Product format categories	Description
Burger	Includes “burgers” and “patties”
Sausage	Includes “sausages,” “bangers” and “wursts”
Meatballs	Includes “meatballs” and “minceballs”
Mince	Includes “mince” and “vegemince”
Smallgoods	Includes “deli slices,” “luncheon meat,” “bacon,” “pepperoni” and “hotdogs”
Strips	Includes “strips,” “tenders,” “schnitzel,” “nuggets,” “dippers,” “bites”
Chunks	Includes “chunks,” “shreds,” “pieces” and “fillets”
Falafel	Includes “falafel” (prepared and dry mixes)
Other	Formats with <5 products which fell outside the other categories Includes: “roast,” “nutmeat,” “koftas,” “skewers”

#### 2.1.2 Product packaging

For each product, claims made on the FOP label were systematically coded and classified into one of three claim types: (i) Nutrition claim; (ii) Health claim; and (iii) Other claim. Claims were further divided into sub-categories, based on their category and content (Table 3). Nutrition claims were further divided into “nutrition content claims” and “health-related ingredient claims”; Health claims were further divided into “general health claims” and “reduction of disease risk claims”; and Other claims were further divided into “environment claims” and “other health-related claims.” These categories and classifications were guided by the taxonomy for health-related food labeling developed by the International Network for Food and Obesity/Non-Communicable Diseases Research, Monitoring, and Action Support (INFORMAS) (40) and adapted to address the aims of the study, and for comparability to other studies (20, 38).

**TABLE 3:** Classification of food-labeling components with examples.

Claim type	Subcategory	Examples
Health claim	General health claim	Health / Healthy Nutritious Low GI
	Reduction of disease risk claim	National Heart Foundation Approved (Tick)
Nutrition claim	Nutrition content claim	**Positive nutrients** High in fiber Good source of protein
		**Negative nutrients** Low saturated fat Cholesterol free
	Health-related ingredient claim	Wholegrain One serve of veggies per burger 100% vegetables
Other claim	Environment claim	Organic Non-GMO
	Other health-related claims	**Dietary pattern** Vegetarian / Vegan Plant-based Meat-free / No meat
		**Positive statement** Natural / Naturally / Nature Wholefood, wholesome Proper food Health Star Rating
		**Highlighted ingredient** Made from soy Packed with veg, vegie-full, vegie burger
		**Free-from statement** No artificial colors, flavors or preservatives Soy-free Free from dairy Gluten free/Wheat free

Adapted from “nutritional marketing of plant-based meat-analog products: an exploratory study of front-of-pack and website claims in the USA”, by J. Lacy-Nichols, L. Hattersley and G. Scrinis, *Public Health Nutrition*, 24(14), p.4434 (20).

When a single claim could be classified as more than one type of claim, a hierarchal ranking was adopted: Health claim > Nutrient claim > Other claim. Where two or more claims were made within one phrase, they were coded as separate claims. For example, “naturally high in fiber” was coded as (i) nutrition content claim (high in fiber), and (ii) other health-related claim (naturally). Where claims were repeated on the FOP label, all repetition was coded individually and included as separate claims. For example, “suitable for vegetarians,” “meat-free” and plant-based” claims on the same label were included as three separate “other health-related” claims.

Promotion of products through their packaging was also recorded. Specifically, the use of transparent packaging (or windows) so products were visible through the packaging was recorded. Imagery of the product as presented on the FOP label, and use of environmental depictions (such as vegetables/nature) was also recorded as alternative text. These images were then categorized broadly into either animal imagery (e.g., cartoons of farm animals), nature imagery (sub-categorized into plants or scenery such as hills or a sun) or images/cartoons of vegetables, legumes and seeds.

#### 2.1.3 Product pricing

Price per serve was calculated from the manufacturers recommended serve size as displayed on the nutrition information panel, and the unit pack size and price:


$$p\,r\,i\,c\,e\,p\,e\,r\,s\,e\,r\,v\,e=\frac{u\,n\,i\,t\,p\,r\,i\,c\,e\,\left(A\,U\,D\right)}{p\,a\,c\,k\,s\,i\,z\,e\,\left(g\right)}\times s\,e\,r\,v\,e\,s\,i\,z\,e\,\left(g\right)$$

#### 2.1.4 In-store product promotion

In 2021, photographs capturing the location of alternative protein products across eight supermarkets were also collected. Images were systematically recorded to collect information regarding (i) the store layout: specifically, sections where alternative protein products were found in-store (such as refrigerated and frozen sections); and (ii) cross-merchandising: the products which share the surrounding shelf-space of alternative protein products. “Sections” were defined by the presence of a refrigerator or freezer wall, or a door to another section of the refrigerator/freezer. Any promotional material (“in-store displays”), such as shelf tags, banners or flags, to identify products as “plant-based” or “vegetarian” were also collected.

### 2.2 Statistics

Data were analyzed using the Statistical Package for Social Sciences (SPSS) version 26 (IBM, New York, USA). Outliers for serve size and price data were identified using minimum and maximum values and by viewing boxplots. These values were double checked by reviewing the original photographs, and corrected if necessary. Products which did not display values for serve size were recorded as missing values. Data were checked for normality using the Shapiro–Wilk test and visual inspection of histograms. Most data were normally distributed; therefore, results were presented as Means and Standard Deviations. Categorical data were described using counts and percentages. Two-way ANOVAs were conducted to explore the impact of product type and collection year on price (per 100 gram and per serve). Statistical significance was set at *p* < 0.05.

## 3 Results

**FIGURE 1:**
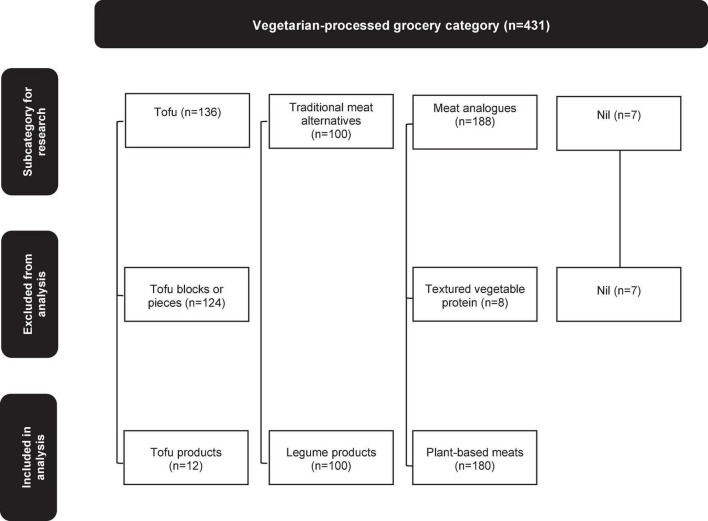
Overview of the Vegetarian-processed grocery category and subcategory of products included in the analysis. Counts are of products collected in 2014, 2017, and 2021.

Data from 431 alternative protein products were collected in the FoodTrack™ database over the 3 years studied, of which 292 met the eligibility criteria for inclusion in the analysis (Figure 1). Sixty-six products were collected in 2014 (23% of products included in the analysis), 74 in 2017 (25%) and 152 in 2021 (52%), showing growth in the category over time. Most were plant-based meats (*n* = 180, 62%), followed by legume products (*n* = 100, 34%), and tofu products (*n* = 12, 4%). Most alternative protein products (*n* = 242, 83%) were branded. The remaining products (*n* = 50) were private-label products, most of which were legume products (*n* = 32). Less than 10% of tofu products (*n* = 1 of 12, 8%) and plant-based meats (*n* = 17 of 180, 9%) were private-label products.

Regarding country of origin, nearly two-thirds of alternative protein products (*n* = 177, 61%) were made in Australia. Others were imported from the United Kingdom (14%), South Africa (6%), and New Zealand (5%), with fewer products imported from North and South America, and European and Asian countries. Country of origin information was not available for two products.

### 3.1 Product format

Across the category of alternative protein products, “burgers” (*n* = 86), “strips” (*n* = 51) and “sausages” (*n* = 42) were the most common product formats, accounting for ∼61% of the product range. All other format categories (“meatballs,” “mince,” “smallgoods,” “chunks,” “falafel” and “other”) each contributed to <10% of the product range.

**FIGURE 2:**
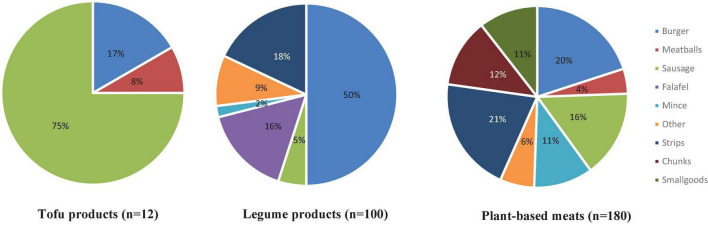
The range and proportion of formats of alternative protein products.

Figure 2 shows the range and proportion of formats among tofu products, legume products and plant-based meats. There was greater variety in the formats available for legume products and plant-based meats than tofu products, which were only available as sausages (*n* = 9, 75%), burgers (*n* = 2, 17%) and meatballs (*n* = 1, 8%). Nearly half of the legume products were prepared as burgers (*n* = 48, 48%) compared with one fifth of plant-based meats (*n* = 36, 20%), and about one fifth of the legume (18%) and plant-based meat products (21%) were presented as strips. Some product formats were exclusive to particular product categories. By definition, falafel were exclusively legume products, and smallgoods and chunks were only available in the plant-based meat category.

Generally, the distribution of product formats was consistent over time, relative to the increased number of products collected over the years (Table 4).

**TABLE 4:** Changes in product numbers and format of alternative protein products sold in 2014, 2017, and 2021.

	Tofu products (*n* = 12)			Legume products (*n* = 100)			Plant-based meats (*n* = 180)			Total
	**2014**	**2017**	**2021**	**2014**	**2017**	**2021**	**2014**	**2017**	**2021**	
Burger (*n*)	2	0	0	13	16	19	9	7	20	86
Chunks (*n*)	0	0	0	0	0	0	4	7	11	22
Falafel (*n*)	0	0	0	3	6	11	0	0	0	20
Meatballs (*n*)	1	0	0	0	0	0	1	0	7	9
Mince (*n*)	0	0	0	0	1	1	4	4	11	21
Sausage (*n*)	2	3	4	2	0	3	7	8	13	42
Smallgoods (*n*)	0	0	0	0	0	0	5	3	11	19
Strips (*n*)	0	0	0	2	6	6	8	8	21	51
Other* (*n*)	0	0	0	1	2	8	2	3	6	22

*“Other” products included roasts, nutmeats, koftas, and dry mixes (such as fritter mix, chicken mix).

### 3.2 Product packaging

#### 3.2.1 Packaging features

One in four (*n* = 113, 39%) alternative protein products had clear transparent packaging (or a transparent window), so the product was visible through the packaging. Use of transparent packaging was more common among legume products (*n* = 62, 62%), compared with plant-based meats and tofu products (*n* = 48, 27%; and *n* = 3, 25%, respectively). Over time, the use of clear transparent packaging generally increased (2014: *n* = 21, 32%; 2017: *n* = 16, 22%; 2021: *n* = 76, 50%).

Most products (*n* = 239, 81%) presented an image of the prepared product on the FOP label, either as a standalone item (e.g., grilled sausages on a plate), or as part of a meal (such as a burger (product) in a bun with salad, served on a plate with chips). Of these, seven (2% of total products) were cartoons, while the remainder were photographs. Plant-based meats displayed product images more frequently than legume or tofu products (*n* = 164, 91%; *n* = 67, 67%; *n* = 8, 67%, respectively). Images of products were more common in 2017 (*n* = 66, 89%), but less common in 2014 (*n* = 46, 70%), compared with 2021.

Other imagery was also used on the FOP label. Twelve (4%) alternative protein products displayed animal imagery (usually a cartoon depiction or cut-out), 11 of which were plant-based meats. The number of products displaying animal imagery on the FOP label was consistent in 2017 and 2021 (*n* = 6 products each year). No animal imagery was used on products collected in 2014. Imagery of vegetables/legumes or seeds (*n* = 56, 19%) and nature (*n* = 139, 48%) were also used. The most common of these was images (often stylised cartoons) of leaves or non-edible plants (*n* = 105, 36%). Cartoon scenery (e.g., mountains, rivers) were also used (*n* = 24, 8%). There was no clear trend regarding the use of vegetable/legume imagery over time (2014: *n* = 16, 24%; 2017: *n* = 5, 7%; and 2021: *n* = 31, 20%), whereas the use of environmental imagery decreased over time (2014: *n* = 39, 59%; 2017: *n* = 35, 47%; and 2021: *n* = 61, 40%).

About a third of plant-based meats collected in 2014 and 2021 (*n* = 29) also contained instructions to guide consumers on either how to cook or consume the product. Some examples were “perfect for pasta,” “use in any chicken recipe” or “just grill it.” Instructions such as these did not appear on the FOP label for any tofu or legume product, nor did they appear on any products in 2017.

#### 3.2.2 Front of pack claims

**FIGURE 3:**
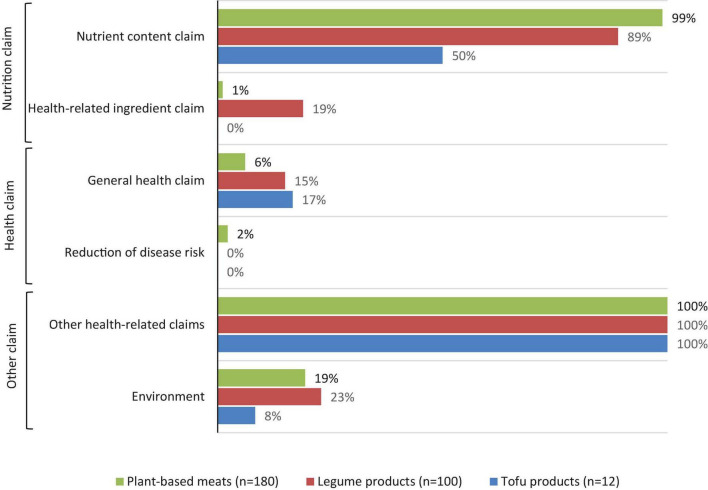
The proportion of alternative protein products displaying nutrition, health, or other claims on their front of pack label.

A summary of claims made on the FOP labels for tofu products, legume products and plant-based meats is illustrated in Figure 3. Within the nutrition related category, claims about the nutrient content were more common than health-related ingredient claims. General health or disease related claims were not common, with less than 10% of products displaying a general health claim. All products contained at least one “other health-related claim,” and approximately 20% of products made an environment-related claim. These claims are described in more detail below.

##### 3.2.2.1 Nutrition claims

###### 3.2.2.1.1 Nutrient content claim

Almost all (99%) plant-based meats made a nutrient content claim, 89% of legume products and 50% of tofu products. “Positive” nutrient claims were used on FOP labels four times more than negative nutrient claims (432 versus 101, respectively). Overall, protein-related claims were the most common “positive” nutrient claim (*n* = 180, 62%), followed by fiber (*n* = 101, 35%), vitamin B12 (*n* = 61, 21%) and iron (*n* = 58, 20%). Claims about zinc and fatty acids occurred less frequently and were made on plant-based meats more commonly than tofu products and legume products (Table 5). “Negative” nutrient claims were related to cholesterol, fat, energy and sugar. Claims about cholesterol (“cholesterol free” and “0 mg cholesterol”) were most common, and appeared on a quarter of products (*n* = 72, 25%).

**TABLE 5:** Content on the front of pack label in 2014, 2017, and 2021.

	Tofu products (*n* = 12)			Legume products (*n* = 100)			Plant-based meats (*n* = 180)		
	2014 (*n* = 5)	2017 (*n* = 3)	2021 (*n* = 4)	2014 (*n* = 21)	2017 (*n* = 31)	2021 (*n* = 48)	2014 (*n* = 40)	2017 (*n* = 40)	2021 (*n* = 100)
Claim type and content	*n* (%)	*n* (%)	*n* (%)	*n* (%)	*n* (%)	*n* (%)	*n* (%)	*n* (%)	*n* (%)
**Nutrition claim**									
Nutrient content claim	5 (100%)	1 (33%)	0	20 (95%)	30 (97%)	39 (81%)	39 (97%)	40 (100)	99 (99%)
**Positive nutrients**									
Protein	5 (100%)	1 (33%)	0	1 (5%)	9 (29%)	17 (35%)	31 (78%)	40 (100%)	76 (76%)
Iron	1 (20%)	0	0	0	3 (10%)	4 (8%)	13 (33%)	20 (50%)	17 (17%)
Vit. B12	1 (20%)	0	0	1 (5%)	2 (6%)	4 (8%)	13 (33%)	20 (50%)	20 (20%)
Zinc	1 (20%)	0	0	1 (5%)	1 (3%)	0	12 (30%)	14 (35%)	1 (1%)
Fiber	2 (40%)	0	0	8 (40%)	13 (42%)	18 (28%)	13 (33%)	17 (43%)	30 (30%)
Fatty acids	0	0	0	0	0	0	0	1 (5%)	1 (1%)
**Negative nutrients**									
Cholesterol	0	0	0	11 (52%)	5 (16%)	1 (2%)	21 (53%)	22 (55%)	12 (12%)
Fat (general)	0	0	0	0	1 (3%)	0	2 (5%)	4 (10%)	2 (2%)
Fat (saturated)	0	0	0	0	0	1 (2%)	0	1 (3%)	10 (10%)
Energy	0	0	0	0	0	0	0	0	6 (6%)
Sugar	0	0	0	0	0	1 (2%)	0	0	1 (1%)
No nutrient content claim	0	2 (66%)	4 (100%)	1 (5%)	1 (3%)	9 (19%)	1 (3%)	0	1 (1%)
Health-related ingredient	0	0	0	6 (29%)	6 (19%)	7 (15%)	1 (3%)	0	1 (1%)
1 serve of veg	0	0	0	6 (29%)	4 (13%)	6 (13%)	1 (3%)	0	1 (1%)
100% vegetable	0	0	0	0	1 (3%)	0	0	0	0
7 veg	0	0	0	0	0	1 (2%)	0	0	0
Wholegrain	0	0	0	0	1 (3%)	0	0	0	0
No health-related ingredient claim	5 (100%)	3 (100%)	4 (100%)	15 (71%)	25 (81%)	41 (85%)	39 (98%)	40 (100%)	99 (99%)
**Health claim**									
General health	1 (20%)	0	1 (25%)	1 (5%)	3 (10%)	11 (23%)	10 (25%)	0	1 (1%)
Low GI	0	0	0	0	1 (3%)	2 (4%)	0	0	0
Nutritious	0	0	0	0	1 (3%)	0	0	0	0
Good for you / Goodness	0	0	0	1 (5%)	0	10 (21%)	0	0	1 (1%)
Health / Healthy / Wellbeing	1 (20%)	0	0	0	2 (6%)	0	10 (25%)	0	0
No general health claim	4 (80%)	3 (100%)	3 (75%)	20 (95%)	28 (90%)	37 (77%)	30 (75%)	40 (100%)	99 (99%)
Reduction of disease risk	0	0	0	0	0	0	3 (8%)	1 (3%)	0
National Heart Foundation Approved (Tick)^†^	0	0	0	0	0	0	3 (8%)	1 (3%)	0
No reduction of disease risk claim	5 (100%)	3 (100%)	4 (100%)	21 (100%)	31 (100%)	48 (100%)	37 (93%)	39 (98%)	100 (100%)
**Other claims**									
Environment-related claim	0	0	1 (25%)	6 (29%)	11 (36%)	6 (13%)	7 (18%)	4 (10%)	24 (24%)
Organic	0	0	0	2 (10%)	1 (3%)	2 (4%)	0	0	0
GMO-free	0	0	0	4 (19%)	10 (32%)	2 (4%)	7 (18%)	4 (10%)	24 (24%)
Palm oil free	0	0	1 (25%)	0	0	3 (6%)	0	0	0
No environment-related claim	5 (100%)	3 (100%)	3 (75%)	15 (71%)	20 (65%)	42 (88%)	33 (83%)	36 (90%)	76 (76%)
Other health-related claim	5 (100%)	3 (100%)	4 (100%)	21 (100%)	31 (100%)	48 (100%)	40 (100%)	40 (100%	100 (100%)
**Dietary pattern**									
Vegan / Vegetarian	3 (60%)	2 (67%)	3 (75%)	14 (67%)	26 (84%)	36 (75%)	33 (100%)	33 (83%)	60 (60%)
Plant-based	0	0	1 (25%)	0	0	16 (33%)	0	0	62 (62%)
Meat-free	0	3 (100%)	0	0	1 (3%)	4 (8%)	20 (61%)	18 (45%)	48 (48%)
Other	0	0	0	4 (19%)	1 (3%)	0	0	0	8 (8%)
No dietary pattern	2 (40%)	0	0	7 (33%)	4 (13%)	6 (13%)	7 (18%)	0	1 (1%)
**Positive statement**									
Wholefoods / Ingredients you know	1 (20%)	0	0	6 (29%)	4 (13%)	6 (13%)	2 (5%)	0	1 (1%)
It’s what’s on the inside that counts	1 (20%)	0	0	0	0	0	7 (18%)	0	0
Better for everyone	0	0	1 (25%)	0	0	3 (6%)	0	0	0
Eat well	0	0	0	5 (24%)	5 (16%)	1 (2%)	0	0	0
Clean	0	0	0	0	0	0	0	0	3 (3%)
Natural / Naturally	0	0	0	4 (19%)	4 (13%)	14 (29%)	0	4 (10%)	10 (10%)
Proper food	0	0	0	0	0	0	0	0	1 (1%)
Health Star Rating*	0	3 (100%)	1 (25%)	0	13 (42%)	25 (52%)	0	24 (60%)	65 (65%)
No positive statement	3 (60%)	0	3 (75%)	6 (29%)	12 (39%)	20 (42%)	33 (83%)	12 (30%)	26 (26%)
**Highlighted ingredient**									
Soy	3 (60%)	1 (33%)	0	0	0	0	0	0	2 (2%)
Pea	0	0	0	0	0	0	0	0	2 (2%)
Vegie / Vegie full / Packed with veg	2 (40%)	0	3 (75%)	7 (33%)	12 (39%)	18 (38%)	1 (3%)	0	2 (2%)
Legumes / Grains	0	0	0	8 (38%)	11 (35%)	18 (38%)	0	0	5 (5%)
No highlighted ingredients	0	2 (67%)	1 (25%)	10 (48%)	12 (39%)	13 (27%)	39 (98%)	40 (100%)	89 (89%)
**Free-from statement**									
Soy-free	0	0	0	0	0	0	14 (35%)	9 (23%)	7 (7%)
Dairy-free	0	0	0	15 (71%)	11 (35%)	9 (19%)	3 (8%)	0	6 (6%)
Lactose-free	0	0	0	3 (14%)	0	0	0	0	0
Gluten-free (or wheat-free)	1 (20%)	1 (33%)	0	6 (29%)	15 (48%)	16 (33%)	0	8 (20%)	8 (8%)
No MSG	0	0	0	0	0	0	1 (3%)	0	2 (2%)
No artificial flavors/Colors/Preservatives	1 (20%)	0	0	0	11 (35%)	16 (33%)	20 (50%)	11 (28%)	18 (18%)
No antibiotics/Hormones/Chemicals	0	0	0	0	1 (3%)	0	0	4 (10%)	0
Egg-free	0	0	0	0	1 (3%)	0	0	0	0
No free-from statements	3 (60%)	2 (67%)	4 (100%)	6 (29%)	11 (35%)	21 (44%)	18 (45%)	19 (48%)	63 (63%)
No other health-related claims	0	0	0	0	0	0	0	0	0

^†^The National Heart Foundation Tick was retired in December (62); *the Health Star Rating was established in June 2014 (63).

There were some changes observed over time; “negative” nutrient claims were less frequently used in 2021 than earlier years (9% of products in 2021 versus 48% in 2014), whereas the use of “positive” claims was more consistent between years. Claims highlighting the presence of zinc decreased over time (21% of products in 2014 versus < 1% in 2021), as did claims highlighting the absence of cholesterol (48% in 2014 versus 9% in 2021). Parallel to this, there was an increase in the number of claims about products’ fat content (9% in 2021 versus 3% in 2014). No tofu products made negative nutrient claims.

###### 3.2.2.1.2 Health-related ingredient

Less than 10% of alternative protein products contained a health-related ingredient claim (*n* = 21, 7%). With the exception of one claim about “wholegrain,” all health-related ingredient claims were related to the presence of vegetables in products. Health-related ingredient claims were almost exclusively found on legume products (*n* = 19 of 21). There were limited changes to the number of health-related ingredient claims over time (Table 5).

##### 3.2.2.2 Health claims

###### 3.2.2.2.1 General health claim

There were four different general health claims made on the FOP labels, which were made on around 10% of alternative protein products (*n* = 28 of 292). Claims including the terms “health,” “healthy” or “wellbeing” were most common (*n* = 13), followed by “good for you,” and “goodness” (*n* = 12). “Low GI” and “nutritious” were used the least (*n* = 3 and *n* = 1, respectively). General health claims were used on 17% of tofu products, 15% of legume products and 6% of plant-based meats. Changes over time were observed for the terms “good for you” or “goodness,” which were more common in 2021 (*n* = 11, 17%) compared with 2014 (*n* = 1, 1%). In contrast, the use of “healthy”/ “health” and “wellbeing” terms decreased over time (2014: *n* = 11, 17%; 2017: *n* = 2, 3%; and 2021: *n* = 0, 0%).

###### 3.2.2.2.2 Reduction of disease risk claim

There was only one type of health claim associated with “reduction of disease risk”; the National Heart Foundation Approved “Tick.” The “Tick” was only found on plant-based meat products in 2014 (*n* = 3, 8%) and 2017 (*n* = 1, 3%). Of note, the “Tick” program was retired in 2015.

##### 3.2.2.3 Other claims

###### 3.2.2.3.1 Environment-related claim

Environment-related claims were made on one fifth of products (*n* = 59, 20%). The most common claims were variations of “not genetically modified” (*n* = 51), whereas claims about “organic” (*n* = 5) and the absence of palm oil (*n* = 4) were less common. Claims about being “organic” were only made on legume products (*n* = 5). Across the range of alternative protein products, the proportion of products with an environment-related claim was consistent over time (2014: *n* = 13, 20%; 2017: *n* = 15, 20%; and 2021: *n* = 31; 20%). Claims about the absence of palm oil were exclusive to products collected in 2021 (Table 5).

###### 3.2.2.3.2 Dietary pattern

Most products (*n* = 265, 91%) mentioned a dietary pattern, or a combination of dietary patterns on their FOP label. Vegan/vegetarian claims were most common (*n* = 210 of 265 products, 79%), followed by meat-free (*n* = 94, 36%), plant-based (*n* = 79, 30%), and other (*n* = 13, 5%) which included halal and kosher. “Plant-based” claims were only made on the FOP label of products collected in 2021.

###### 3.2.2.3.3 Positive statement

Nearly two-thirds (*n* = 177, 61%) of alternative protein products made “positive statements,” which appeared relatively evenly across tofu products (*n* = 6 of 12, 50%), plant-based meats (*n* = 109 of 180, 61%) and legume products (*n* = 62 of 100, 62%). “Wholefoods” and “natural” / “naturally” claims were more commonly used on legume products than tofu products and plant-based meats (Table 5). The Health Star Rating (established in 2014) was displayed on approximately half of alternative protein products (*n* = 131, 45%), only on products collected in 2017 and 2021. Ten of the 131 products (8%) had a Health Star Rating of 3 or fewer stars (range 1 to 5 stars, average 4.1 ± 0.6 stars; data not shown). The Health Star Rating was more commonly found on plant-based meats (49%) than legume products (38%) and tofu products (33%).

###### 3.2.2.3.4 Highlighted ingredient

About a third (*n* = 86, 29%) of alternative protein products made one or more highlighted ingredient claims on the FOP label. “Highlighted ingredients” were predominantly found on legume products (76%, compared with 13% on plant-based meats and 10% on tofu products) and related to the presence of vegetables and legumes (Table 5). In 2021, soy was a highlighted ingredient (“made from soy”) on two plant-based meat products.

###### 3.2.2.3.5 Free-from statement

Approximately half the alternative protein products made at least one “free-from” statement (*n* = 145, 50%). “No artificial colors, flavors or preservatives” (or variations) was the most common “free-from” statement, which featured on about a quarter of products (*n* = 77, 26%), followed by gluten- (or wheat-) free (*n* = 55, 19%) and dairy-free (*n* = 44, 15%). “Soy-free” claims were made exclusively on plant-based meat products, and more commonly used in 2014 (35%) and 2017 (23%) compared with 2021 (7%). A similar trend was seen for “dairy-free” claims on legume products: 71% of products in 2014 compared with 19% in 2021.

### 3.3 Product pricing

Overall, the unit price of alternative protein products ranged between $2.50 and $15.00, and the price per serve ranged from $0.44 to $5.50. The serving size and price of alternative protein products are available in Table 6.

**TABLE 6:** Serving size and price (AUD) of alterative protein products collected in 2014, 2017, and 2021.

		2014	2017	2021	Δ 2014 to 2021
**Size**					
Pack size (g)	Tofu products	294.0 (85.9)	300.0 (0.0)	281.3 (62.5)	−4%
	Legume products	368.6 (107.4)	304.7 (104.4)	261.0 (67.6)	−29%
	Plant-based meats	303.3 (75.3)	297.7 (123.1)	274.7 (77.1)	−9%
Serving size (g)	Tofu products	123.0 (43.0)	116.7 (28.9)	125.0 (0.0)	+2%
	Legume products	88.6 (40.7)	93.9 (30.4)	98.5 (30.5)	+11%
	Plant-based meats	85.1 (22.8)	84.6 (20.3)	89.0 (29.4)	+5%
**Price**					
Unit price ($)	Tofu products	6.1 (1.7)	7.8 (0.8)	6.5 (1.0)	+7%
	Legume products	5.2 (1.3)	5.1 (1.3)	5.5 (1.2)	+6%
	Plant-based meats	6.1 (1.1)	6.6 (1.7)	7.0 (1.8)	+15%
Price per 100 g ($)	Tofu products	2.1 (0.2)	2.6 (0.3)	2.3 (0.1)	+12%
	Legume products	1.5 (0.7)	1.8 (0.6)	2.2 (0.6)	+42%
	Plant-based meats	2.1 (0.6)	2.4 (0.8)	2.8 (1.0)	+28%
Price per serve ($)	Tofu products	2.6 (0.9)	3.0 (0.4)	2.9 (0.2)	+14%
	Legume products	1.1 (0.4)	1.6 (0.5)	2.1 (0.8)	+86%
	Plant-based meats	1.8 (0.7)	2.0 (0.6)	2.3 (0.9)	+31%

Prices are in AUD and are the regular sale price at the time of collection (i.e., not a promotional price). Data presented as mean (SD). Where *n* = 1, data shown are for the single product; Price per serve was calculated based on manufacturers recommended serve size listed on pack.

Tofu products had the largest recommended serving size (122.1 ± 28.9 grams), which was greater than that of both legume products (95.0 ± 32.7 grams, *p* = 0.007) and plant-based meats (87.2 ± 26.1 grams, *p* < 0.001). Subsequently, tofu products were the most expensive per serve ($2.80 ± 0.6), and were more expensive than plant-based meats ($2.12 ± 0.8, *p* = 0.007), and legume products ($1.75 ± 0.7, *p* < 0.001). In contrast, plant-based meats were most expensive per 100 grams ($2.54 ± 0.9) and were significantly more expensive than legume products ($1.94 ± 0.7, *p* < 0.001). The price of tofu products ($2.30 ± 0.3) was not significantly different from the other subcategories.

**FIGURE 4:**
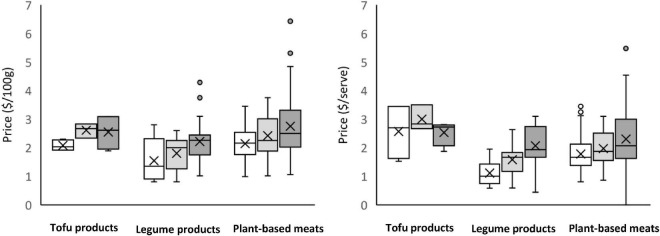
Price of alternative protein products in 2014 (unshaded), 2017 (light shading) and 2021 (dark shading). **(Left)** Price per 100 g; **(Right)** price per serve. ALDI and IGA products included in the dataset from 2017. Prices are in AUD and are the regular sale price at the time of collection (i.e., not a promotional price). Price per serve was calculated based on manufacturers recommended serve size as listed on pack.

Between 2014 and 2021, on average, the pack size decreased (14% decrease) and the unit price increased (9% increase). There was a significant main effect for collection year (price/100 g, *p* = 0.035; price/serve, *p* = 0.003); products were more expensive in 2021 compared with both 2014 and 2017. The interaction effect (product type by collection year) did not reach statistical significance, whether prices were expressed as per 100 gram or per serve. The price of alternative protein products collected in 2014, 2017, and 2021 is illustrated in Figure 4.

### 3.4 In-store product promotion (2021 only)

#### 3.4.1 Store layout

Alternative protein products collected in the FoodTrack™ database appeared in 21 locations across the eight stores visited in 2021 (range 2 – 4 locations per store, mean = 2.6). Alternative protein products were typically displayed in one section of the freezer department and either one or two refrigerated areas. On average, there was a greater proportion of plant-based meats found in the freezer department (60%) compared with tofu products and legume products (6% each). Table 7 presents the location of alternative protein products across the eight supermarkets visited.

**TABLE 7:** Location of products from each category within eight different supermarket locations.

	% Of products displayed in each location					
	**Tofu products**	**Legume products**	**Plant-based meats**	**Products sharing the section/Display**	**Products in neighboring sections**	**Section/Shelf labels or flags to indicate range?**
**Freezer**						
Store A-1	0	0	80%	Ready meals	Ready meals, frozen vegetables	No (“meals”)
Store A-2	0	0	80%	Potato gratin products	N/A – endcap	No
Store B-1	0	6%	51%	Gluten-free products (ready meals), savory party snacks	Savory party snacks (pies, sausage rolls), gluten-free products (pastry, pasta, garlic bread)	No
Store B-2	33%	0	54%	Gluten-free products (crumbed chicken, pastry, pizza bases, ready meals, garlic bread, savory party snacks)	Ready meals, frozen vegetables	Meat-free monday, any night!” (sticker)*
Store C-1	0	40%	91%	Savory party snacks	Savory party snacks (dumplings, spring rolls)	“Make it meat free” (shelf tag)*
Store C-2	0	6%	78%	Gluten-free products (ready meals, crumbed chicken, pizza bases, pastry)	Gluten free products (conventional meat products – honey chicken, sweet and sour pork, fried rice), Frozen desserts	“Make it meat free” (shelf tag)*
Store D-1	0	0	52%	Exclusively meat-alternatives	Ready meals, frozen vegetables	No (“vegetables”)
Store D-2	0	6%	41%	Exclusively meat-alternatives	Conventional meat - battered fish, Frozen vegetables	“Vegetarian and plant based” (sticker)
Average	6%	6%	60%			
**Chilled – general**						
Store A-1	0	100%	20%	Fresh pasta, Tofu, Ready meals, Pizza	Conventional meat – sausages and smallgoods, Juices	No (“ready meals”)
Store A-2	0	100%	20%	Fresh pasta, Tofu, Ready meals, Pizza	Pre-made salads, juices	No
Store B-1	100%	94%	19%	Tofu, other plant-based products (dressing/sauces, desserts), liquid egg whites, Fermented cabbage products	Packaged seafood (smoked salmon, prawns, mussels)	No
Store B-2	67%	100%	22%	Cheese + crackers, fermented cabbage products	N/A – endcap	No
Store C-1	–	–	–	–	–	–
Store C-2	100%	94%	22%	Tofu, vegan products (cheese, dressings/sauces, probiotic drinks), bone broth (free-range chicken bone broth, grass-fed free-range beef bone broth), protein smoothie	Frozen meals, frozen bakery products, plant-based products, including pesto, sauerkraut, protein smoothies, vegan cheese	”Plant-based destination” (shelf label)
Store D-1	0	70%	48%	Vegan products (cheese, sour cream), Tofu, dressings and sauces, fermented cabbage products	Conventional meat - smoked salmon, prawns, octopus	”Enjoy alternative plant-based foods and meals. Perfect for meatless monday” and “ready to enjoy non-dairy. Try our range
						of delicious milk alternatives like Soy, Almond and Coconut”
Store D-2	0	50%	0	Vegan products (cheese, sour cream), dressings/sauces, Fermented cabbage products	Dairy products (cheese)	No
Average	33%	79%	12%			
**Chilled – meat section**						
Store A-1	–	–	–	–	–	–
Store A-2	–	–	–	–	–	–
Store B-1	0	0	30%	Conventional meat – kangaroo sausages	Conventional meat sausages, kangaroo products	No (“sausages,” “game”)
Store B-2	0	0	11%	Conventional meat – kangaroo sausages and burgers, organic chicken meat	N/A – endcap	No (“organic,” “game”)
Store B-2	0	0	14%	Conventional meat – sausages	Conventional meat – sausages, sliders	“Meat free” (flag)
Store C-1	100%	60%	9%	Vegan products (cheese, sour cream, sauces), Fresh pasta, Tofu, Fermented cabbage products	N/A endcap	“Vegan and vegetarian” (sign on fridge)
Store C-2	–	–	–	–	–	–
Store D-1	100%	30%	0	Exclusively meat-alternatives	Conventional meat – crumbed chicken products	“Plant-based” (x3 flags)
Store D-2	100%	44%	59%	Exclusively meat-alternatives	N/A – endcap	“Plant-based” (flag)
Average	61%	15%	29%			

*Indicates promotional material was associated with brand-specific campaigns that were running at the time of data collection.

#### 3.4.2 Cross-merchandising

In some cases, alternative protein products were placed in their own section (4 locations, 1 retailer). More commonly, alternative protein products shared shelf space with other food and beverages (17 of 21 locations), however this varied between retailers and between stores.

Most refrigerated products shared shelf space with other plant-based products, such as dairy-free cheeses, sauces and traditional meat alternatives such as tofu blocks (5 of 13 locations; 2 stores, 1 retailer). In three locations, refrigerated alternative protein products (all plant-based meats) shared shelf space with conventional meat products, such as sausages and burgers. In other locations, alternative protein products were positioned with ready meals, fresh pasta, savory snack combinations such as cheese and crackers, or positioned on their own.

There was more consistency (across stores and retailers) for frozen products, which were usually positioned with frozen vegetables, ready meals and savory party snacks (Table 7).

#### 3.4.3 In-store displays

Promotional materials presented alongside the product range to indicate products were of plant origin were displayed in 10 of the 21 locations (48%) across the eight stores (in three out of four retailers). Promotional material was more common in the “chilled meat” sections (4 out of 6) than the frozen (4 out of 8) and general refrigerator (2 out of 7) sections. Promotional material included banners above the refrigerator/freezer or shelf, flags sticking out into the aisle, flat stickers on refrigerator/freezer doors, signs sitting on the top of low-lying refrigerators and flags within the refrigerator/freezer. Messages were mostly generic, indicating that the products were “plant-based,” “vegan and vegetarian” or “meat-free” (Table 7).

## 4 Discussion

Marketing, such as through product descriptors, price, and packaging, plays an important role in consumers’ perceptions and acceptance of new foods. The purpose of this study was to explore the marketing strategies used for alternative protein products in Australia, and investigate whether these marketing strategies have changed over time. There has been growth in this grocery category, particularly for plant-based meats. Plant-based meats and legume products have also innovated more than tofu products, to now have a greater variety of product formats available to consumers, such as burgers, strips and sausages. These formats are familiar to consumers which is important in overcoming neophobia consumers may have toward purchasing new products (41, 42). Further to this, most products presented an image of what the product looks like once prepared, and the use of clear packaging has increased over time allowing consumers to see the product prior to purchasing, again increasing the sense of familiarity. It appears from this analysis that considerable effort has gone into providing consumers with a level of comfort prior to purchasing these alternative protein products.

Generally, manufacturers of these products seem to have focused on the known barriers associated with consumer acceptance to market their products. Low levels of acceptance for alternative protein products have been associated with lower perceived product quality (e.g., more artificial and less natural) and perceived health concerns (particularly regarding lack of protein) compared with conventional meat (22, 23, 43–45). About a quarter (26%) of products included in this analysis highlighted the absence of artificial colors, flavors and/or preservatives, and nearly two-thirds (62%) contained at least one protein-related claim. Plant-based meats also included positive nutrient claims about iron, vitamin B12, zinc and fatty acids; nutrients commonly critiqued in the context of vegetarian and vegan diets (46). Interestingly, the number of cholesterol-related claims decreased over the years. Consumer research suggests cholesterol claims are favored by older adults (47), who may not be the target audience of these convenience alternative protein products in recent years. In contrast, fiber content claims were the most common nutrient content claim made on legume products, along with highlighted ingredients such as vegetables and legumes/grains, and positive statements associated with “wholefoods” and “natural.” This suggests that, within the range of alternative protein products, manufacturers adopt different marketing strategies, clearly distinguished by the type of product and target consumer. Plant-based meats may be more appealing to flexitarians (those who occasionally include conventional meat), whereas vegetarians may prefer products that are less like conventional meat, such as legume products (22, 23).

Another marketing strategy employed by food manufacturers to appeal to consumers is to highlight the palatability of products (48). In this study, most products were presented in formats familiar to consumers. Burgers and patties were most common, aligning with consumer purchasing behavior. According to a report published by the Good Food Institute, the top-selling categories of plant-based meats in the US were burgers, sausages and hot dogs, and patties (49, 50). Illustrations of prepared products were also commonplace on FOP labels. Most (81%) products contained an image on the FOP label to communicate the products’ format and intended use. In addition to the contents on the FOP label, consumers may also be unconsciously influenced by other factors, such as the positioning of alternative protein products in retail food environments. Bacon and Krpan (51) tested whether placing plant-based dishes in a separate “vegetarian section” on restaurant menus (versus incorporating them in a single list with dishes containing conventional meat) influenced consumer choice. Diners who received the menu with a vegetarian section were 56% less likely to order those dishes. The authors hypothesize this result may be due to diners dismissing the vegetarian section as “for someone else” (i.e., vegetarians), or priming people with negative associations about vegetarian food, such as less tasty or nutritious. In this study, positioning alternative protein products with conventional meat equivalents occurred infrequently (*n* = 3 of 21 locations). More commonly, alternative protein products shared space with other vegetarian products (such as non-dairy cheeses and tofu blocks) or alongside convenience products (such as ready meals, party snacks and frozen vegetables), suggesting these products are promoted as convenience foods, or options for individuals with special dietary needs.

An obvious way to distinguish alternative protein products from conventional meat products may be to include dietary pattern claims. In this study, more than 90% of products included a dietary pattern on their FOP label to indicate their plant origin. Vegetarian and vegan claims were the most used dietary pattern on FOP labels. Despite the term being devised more than four decades ago (52), “plant-based” claims were only recorded on products collected in 2021. The emergence of plant-based claims on FOP labels seems inconsistent with findings from consumer research. During a 10-week field experiment, Rosenfeld et al. (53) tracked more than 165,000 consumer decisions to investigate how different dietary pattern claims influenced consumer purchasing behavior of alternative protein foods from a restaurant menu. Items marketed as “vegan” or “vegetarian” were 24% more likely to sell than when the same items were marketed as “plant-based.” These findings are consistent with other work by Anderson et al. (47) who assessed differences in how terms are perceived using a series of pairwise comparisons between labels. When compared to other terms, “plant-based” was least preferred, and vegan was clearly favored over plant-based, although, “feel-good” was the most favored. The authors hypothesize that familiarity and clarity may contribute to this preference, such that consumers are familiar with what it means for food to be vegetarian or vegan, but the term “plant-based” may be less familiar, and poorly defined.

Price is another key aspect influencing consumer purchasing behavior. In a survey of 660 consumers and nutrition professionals in Australia, respondents were asked about their willingness to pay for alternative protein products. Although it varied by product format, most respondents were willing to pay $2.00-$3.00 (AUD) per 100 grams for alternative protein products, which is slightly higher than the average price of conventional meat-based products (3). This aligns with the average price per 100 grams of alternative protein products included in this study ($2.33 ± 0.9). The price of alternative protein products changed over the years studied. Between 2014 and 2021, on average, the price (/100 grams) increased by 32%, at an average rate of 4.6% per year. This is greater than the average increase in the Australian consumer price index from 2014 to 2021 (∼2%) (54), suggesting these products were relatively more expensive in 2021 than 2014. Despite this, findings from a recent pricing analysis of Dutch supermarkets suggests the price gap between conventional meat and alternative protein products has significantly narrowed over the past 5 years (55). However, these products are still considered expensive, and their high cost may be a barrier to some potential consumers (22, 23, 44, 56).

A strength of this study was the use of the FoodTrack™ database, enabling a comprehensive comparison of products collected from Australian supermarkets over multiple years. The supermarket and grocery industry in Australia is highly concentrated, with more than 80% of revenue shared between four companies—Woolworths 37.4%, Coles 28.4%, ALDI 10.5% and Metcash (owns IGA) 7% (57). However, prior to 2016, products from ALDI were not included in the database, and prior to 2017, IGA products were not included. The total number of products available in and after 2017 is likely to have been influenced by the addition of the two stores. As well as becoming more commonplace in supermarkets, meals made with plant-based meats also feature on the menu of restaurants and fast food outlets (49). However, this research was limited to products sold in supermarkets, so the marketing strategies of other food environments remains unknown. Another strength of this study is the clear definition of alternative protein products and their subcategories, which was easy to operationalise. However, there is no standardized definition of meat and non-meat protein replacements (43), therefore the categorisation used herein may not be comparable to other studies. This study included more traditional products such as falafel, and processed legume products which other studies have excluded from their analyses. This allowed for a more comprehensive comparison of the alternative protein product market. Finally, the use of the taxonomy for health-related food labeling developed by the International Network for Food and Obesity/Non-Communicable Diseases Research, Monitoring, and Action Support (INFORMAS) was another strength of this study. This approach enables standardized comparisons of product labeling in different countries, and across different retail outlets and food categories. However, the taxonomy does not differentiate between claims which are regulated by Food Standards Australia New Zealand (58), versus unsubstantiated claims.

Marketing has been shown to influence consumer awareness and purchase of food products. This study focused on the marketing strategies used on alternative protein products, but their influence on the purchasing habits of Australian consumers was out of scope. This study is also limited to claims made on the FOP label. Often other information is included on back of pack labels, such as packaging information (e.g., eco-friendly, or recycling instructions) and cooking instructions. The “prominence” of terms on the FOP label (such as font styles and sizes and frequency of terms) were not recorded, nor was the color of product packaging. The location of products in supermarkets was only collected in 2021. Therefore, it is unknown if retailers have changed the location of products over time, particularly as the product range increased. The location of alternative protein products differed between retailers, but also across different locations with the same retailer. It is plausible that the location of products may have been influenced by the store size, i.e., stores with larger floor space may have “more room” to separate products in their own section. However, details about the store size were not collected. Finally, data describing the prevalence of the National Heart Foundation “Tick” and Health Star Rating on product labels is limited. The “Tick” was retired in 2015, and, parallel to this, the Health Star Rating was developed (in 2014). Therefore, comparing the prevalence of the use of these logos over time should be interpreted with caution.

Individuals are shifting toward more plant-based dietary patterns. Consequently, the demand for, and availability of, alternative protein products is growing globally. Marketing plays an important role in consumers’ food related perceptions, acceptance and purchase decisions. This study provides a comprehensive overview of the marketing strategies used by manufacturers and retailers to promote alternative protein products, using the marketing mix as a framework. Consumer interest changes over time, influenced by societal, health, environmental and socioeconomic factors (59). In recent years, many consumers are looking to buy more local, sustainable “clean label” products (60). In Australia, there is currently no mandatory regulatory framework prohibiting the use of conventional meat product descriptors (such “burger” and “mince”) on alternative protein products. Recently, the Alternative Proteins Council suggested labeling nomenclature for meat-alternative products in Australian and New Zealand marketplaces (61). These voluntary guidelines reflect emerging international norms for on-pack product labeling successfully implemented across large markets including the US and UK, to promote consistency in labeling across the category both domestically and for international exports. As the market for alternative protein products continues to grow and evolve, it will be important to monitor how these products are marketed. This study provides a useful evidence base to understand the marketing strategies applied to alternative protein products between 2014 and 2021, and act as a baseline for future research investigating the adoption of voluntary regulatory guidelines. Future research may also consider the influence of these marketing strategies on consumer purchasing behaviors, such as through the use of eye-tracking software.

## Data availability statement

The datasets presented in this article are not publicly available for commercial reasons. Requests to access the datasets should be directed to PB, paige.brooker@csiro.au.

## Author contributions

PB conceptualized the study. PB, GH, RW, and MC developed the study design. TP collected the data. PB, KA, and RW prepared the data for analysis. PB and KA analyzed the data. PB was responsible for drafting the manuscript with contribution from KA. All authors contributed to the interpretation of the results and reviewed and approved the final manuscript.
